# Continuous Glucose Monitoring in Healthy Adults—Possible Applications in Health Care, Wellness, and Sports

**DOI:** 10.3390/s22052030

**Published:** 2022-03-05

**Authors:** Roman Holzer, Wilhelm Bloch, Christian Brinkmann

**Affiliations:** 1 Institute of Cardiovascular Research and Sport Medicine, German Sport University Cologne, 50933 Cologne, Germany; roman.holzer@protonmail.com (R.H.); w.bloch@dshs-koeln.de (W.B.); 2 Department of Fitness & Health, IST University of Applied Sciences, 40223 Düsseldorf, Germany

**Keywords:** continuous glucose monitoring, CGM, wearable, application, health care, lifestyle, nutrition, healthy adults, sports, physical activity

## Abstract

Introduction: Continuous glucose monitoring (CGM) systems were primarily developed for patients with diabetes mellitus. However, these systems are increasingly being used by individuals who do not have diabetes mellitus. This mini review describes possible applications of CGM systems in healthy adults in health care, wellness, and sports. Results: CGM systems can be used for early detection of abnormal glucose regulation. Learning from CGM data how the intake of foods with different glycemic loads and physical activity affect glucose responses can be helpful in improving nutritional and/or physical activity behavior. Furthermore, states of stress that affect glucose dynamics could be made visible. Physical performance and/or regeneration can be improved as CGM systems can provide information on glucose values and dynamics that may help optimize nutritional strategies pre-, during, and post-exercise. Conclusions: CGM has a high potential for health benefits and self-optimization. More scientific studies are needed to improve the interpretation of CGM data. The interaction with other wearables and combined data collection and analysis in one single device would contribute to developing more precise recommendations for users.

## 1. Introduction

Regular blood glucose monitoring is a daily and lifelong task required for people with diabetes mellitus to manage their disease and prevent complications [1]. For a long time, blood glucose concentrations could only be determined using laboratory methods [2]. The high specificity and sensitivity as well as the wide measurement range still qualify laboratory analysis as the gold standard for glucose measurement [3]. At present, glucose meters and dry reagent test strips are primarily used for self-monitoring of blood glucose (SMBG), both of which have a lower accuracy than the laboratory methods [3]. They only require a small drop of blood for the measurement [4]. The American Diabetes Association recommends multiple measurements daily for the assessment of glucose levels, especially for patients receiving insulin therapy [5]. However, pricking several times a day is painful and can cause other problems, such as infections or loss of sensitivity due to scarring and callus formation [6].

Over the past 20 years, the disadvantages of SMBG have led to the development of continuous glucose monitoring (CGM) measurement systems [7]. These CGM systems can be easily worn on the body, automatically and constantly measuring the glucose concentration in the interstitial fluid (ISF). The glucose concentration in the ISF of the subcutaneous tissue closely correlates with the glucose concentration in the blood, is pH-stable, and not susceptible to contamination [8,9,10]. However, current CGM systems used for detecting glucose in the ISF have an inevitable time delay, which is especially relevant under rapidly changing glucose conditions (e.g., during exercise [11]), as the diffusion of glucose requires some time [12,13,14].

The CGM sensors are attached to the upper arm or abdomen and can be worn for up to two weeks, depending on the type of system. In some models, the CGM system’s sensor is implanted under the skin and remains there for up to 180 days [15]. The measured glucose value is sent to an output device, usually a handheld reader or smartphone. Some devices automatically alert the user in case of clinically critical values. The presentation of the glucose profile over a longer period provides the user with a more in-depth insight into their glucose profile.

Novel devices currently under development analyze glucose in the ISF using a minimally invasive, microneedle-based technique with a needle penetration depth that is lower than that in conventional CGM devices [16]. Several reviews have already addressed the development of novel glucose-monitoring systems and describe the different measurement techniques in detail [2,3,7,17].

The main metric for assessing the accuracy of CGM systems is the mean absolute relative difference (MARD) [18,19]. Studies showed that the MARD value for several commercially available CGM systems fulfills international accuracy standards [20,21,22,23,24]. Potential differences between blood glucose values and CGM values must always, however, be expected.

One major advantage of CGM systems versus SMBG is that monitoring occurs automatically, meaning glucose can even be monitored in situations when SMBG is not possible or limited. Glucose trends can be visualized using CGM systems. This can be important, especially in situations in which glucose levels change rapidly, for example, during or after food ingestion or exercise.

When interpreting CGM data and glucose dynamics, it is important to remember that many factors affect glucose levels (e.g., food intake (food composition, glycemic load, and timing), exercise (type, duration, intensity, and timing), stress, metabolic conditions (e.g., skeletal muscle insulin sensitivity)). For more in-depth information on how these factors can affect glucose dynamics and on limits of CGM data interpretation, please see existing reviews on this topic [25,26].

Due to their simple application and continuous recording of glucose values, CGM systems are attracting increasing attention from healthy, nondiabetic individuals and from medical research. Although the fundamental glucose reactions to food intake, sports, and stress have been carefully investigated in patients with diabetes mellitus, glucose dynamics and their relevance for healthy individuals and/or athletes are still widely unknown.

This perspective provides an overview of potential CGM applications in healthy individuals for health care, wellness, and sports. Moreover, current challenges and research interests are identified and future prospects addressed.

## 2. Possible Applications

**Figure 1:**
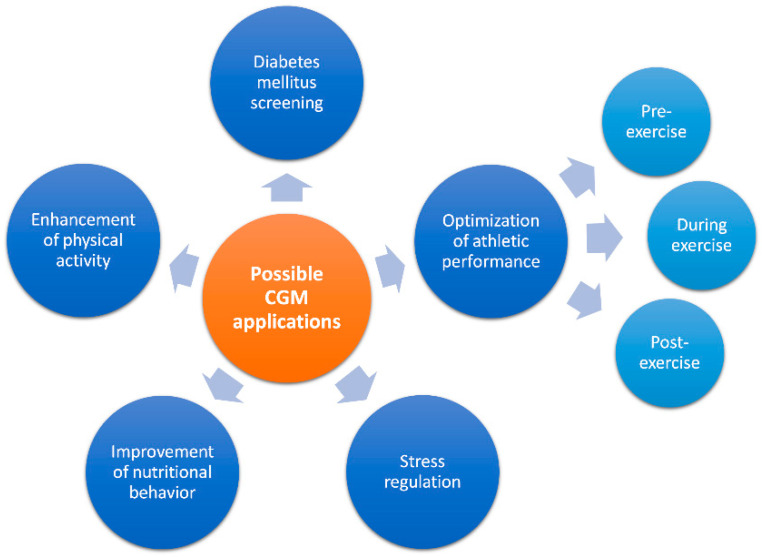
Overview of different potential continuous glucose monitoring (CGM) applications in healthy adults.

In the following section, potential CGM applications beyond diabetes mellitus management are introduced and discussed (Figure 1).

In a recently published multicenter study, CGM systems were used to collect data from healthy, nondiabetic, normal-weight individuals of different age groups [27]. The results showed that the time in range (TIR) from 70 to 140 mg/dL for this population group (n = 153) was 96%, and the mean 24 h glucose was 99 ± 7 mg/dL (5.5 ± 0.4 mmol/L). The American Diabetes Association (ADA) recommends a TIR of >70% for people with diabetes [28]. Values of <70 mg/dL (on average 15 min per day) and >140 mg/dL (on average 30 min per day) were uncommon. These results are in line with previous study outcomes [29,30] and are important for an orientation of CGM data recorded in healthy individuals.

### 2.1. CGM as a Screening Tool for Early Detection of Abnormal Glucose Regulation/Diabetes Mellitus

The diagnosis of prediabetes or diabetes mellitus often relies on fasting glucose and insulin measurements, or on glycated hemoglobin (HbA1c) values, without paying heed to glucose dynamics [31].

Hyperglycemic conditions result in increased production of reactive oxygen species (ROS), resulting in increased oxidative stress and chronic inflammation [32,33]. The oxidative and inflammatory conditions due to hyperglycemia are important risk factors of the development of several diseases such as diabetes mellitus and cancer, as well as cardiovascular complications [33].

CGM data could be useful for early detection of abnormal glucose control and diabetes mellitus, as well as the prevention of oxidative stress and chronic inflammation. In a study on overweight adolescents (n = 118), those with prediabetes had significantly higher mean glucose values, a higher glucose area under the curve (AUC), higher peak glucose values, and longer periods in glucose ranges above 140 mg/dL than adolescents with normal HbA1c or a non-pathological oral glucose tolerance test result [34]. Using CGM data and the derived glycemic variability indices, healthy individuals could be distinguished from those with impaired glucose tolerance or diabetes mellitus with an accuracy of 91.4% [35]. Another research group developed models for identifying potentially impaired glucose regulation [31]. For this purpose, CGM systems were used to record the individual rise in postprandial glucose levels. The specific patterns of the glycemic response led to the subdivision into “glucotypes”. Thereby, the group was able to underline the importance of glucose dynamics in abnormal glucose regulation diagnostics.

### 2.2. CGM for Lifestyle Optimization

Reviews on the use of wearable technology such as fitness trackers in lifestyle interventions have shown that they can be beneficial tools in improving body mass index (BMI) and waist circumference in overweight or obese individuals and increase their motivation for physical activity [36,37,38]. Interventions can be more closely tailored to the individual person through the use of mobile apps [39].

The real-time presentation of glucose values can be effective in inspiring a healthier lifestyle and expanding the field of CGM wearables for lifestyle optimization (including for healthy individuals). Potential lifestyle applications may improve dietary habits, increase physical activity, and improve stress regulation. Some of these beneficial lifestyle changes through the use of CGM have already been observed in patients with prediabetes and diabetes mellitus [40,41].

#### 2.2.1. Nutritional Behavior

CGM studies in healthy but overweight or obese free-living individuals (n = 23) have shown differences in total glucose AUC after meals with high versus low glycemic load [42]. The mean total glucose AUC of a low glycemic load diet was significantly lower compared to that of a high glycemic load diet. CGM systems are therefore able to present different glucose responses to foods with different glycemic loads. This function may be used to develop better awareness of foods and their glycemic load, which in turn may lead to changes in individuals’ nutritional behavior.

This is in line with the findings of a large-scale study from the United States, which investigated the glucose profile of healthy adults and those with prediabetes and non-insulin-treated type 2 diabetes mellitus (n = 665), who all used CGM in combination with a smartphone-based app [43]. Even the short-term 10-day use of CGM significantly improved TIR in the entire group. Young healthy adults showed improvements in TIR more often than the high-glycemic, insulin-resistant patients. Real-time feedback and increased awareness of the effects of various foods on glucose levels appear to be helpful in modifying nutritional behavior, which results in avoiding foods with very high glycemic loads and energy. A regular positive energy balance, which means higher energy uptake than consumption, leads to increased body weight and associated risks of metabolic and cardiovascular diseases in the long term.

In general, low blood glucose concentration is associated with an increase in the feeling of hunger [44]. However, appetite and food intake are also related to other factors such as social interaction, habituation, and emotions [45]. Eating without feeling hungry may result in higher energy uptake than required, long-term body weight gain, and possible obesity-induced complications. Comparing individual sensations with CGM data may be helpful in more closely following the natural feeling of hunger. In “hunger training”, the subjective feeling of hunger can be checked against the glucose concentration displayed on the CGM system. An interventional study showed a positive effect of CGM use in “hunger recognition training”, leading to weight reduction in individuals with obesity (n = 20) [46].

#### 2.2.2. Physical Activity

In addition to a balanced diet and weight management, physical activity is an essential part of a healthy lifestyle. Regular physical activity has a significant impact on health and prevents numerous chronic diseases such as cardiovascular diseases, metabolic diseases, hypertension, cancer, and depression [47,48,49,50,51,52].

High sedentary behavior is linked to increased fasting glucose concentrations, impaired glucose metabolism, and decreased glucose tolerance [53]. Physical activity is an effective way to decrease glucose values and, in particular, postprandial glucose excursions [54,55,56,57,58,59]. Postprandial hyperglycemia was shown to be associated with cardiovascular diseases [54]. The direct feedback and visualization of success (positive effect of physical activity on glucose dynamics) may be a motivating factor for increasing regular physical activity [60,61]. Bailey et al. [60] evaluated the efficacy of an 8-week exercise program using CGM compared to a standard exercise program in terms of exercise adherence among individuals with impaired glucose tolerance (n = 13). Although both groups improved their health-related quality of life, waist circumference, and fitness, the CGM group showed significantly higher increases in program attendance and registration rates for additional exercise programs than the control group. In the investigation by Liao et al. [61], CGM systems were used in combination with fitness trackers in healthy, nondiabetic, overweight subjects (n = 19) during a free-living 10-day period to assess their influence on exercise motivation and physical activity. The results of the study indicated that the use of CGM systems (in combination with a fitness tracker) can support users in transitioning from the precontemplation stage (no intention of changing behavior) to the action stage (changing behavior). The acceptability score for using the CGM system was similar to that of the fitness tracker used during the intervention. The authors observed similar results in another study involving healthy individuals (n = 30), in which the participants also showed a high level of acceptance of CGM and rated its use as simple, useful, and interesting [62].

#### 2.2.3. Stress

Blood glucose levels change not only during food intake and exercise, but also under stress conditions. Increased release of stress hormones leads to a rise in blood glucose concentration. Chronically elevated concentrations of adrenaline and cortisol increase the risk of pathological conditions such as insulin resistance, diabetes mellitus, dyslipidemia, and hypertension [63,64,65].

Continuous glucose monitoring may reveal increases in glucose concentrations that occur independent of food intake or exercise. Real-time presentation of stress-induced glucose responses can help the user take effective stress-reducing actions, such as relaxation practices, to prevent chronic stress conditions.

Interpreting glucose concentrations to analyze stress based only on glucose data is extremely difficult, as glucose regulation depends on many different factors, as explained above. In this regard, many variables would have to be considered, such as time interval since the last meal, its glycemic load, physical activity, etc. However, as the development of sensors to detect cortisol concentrations in the human body is also progressing [66], additive information may lead to more reliable interpretations. This is an example of how integration of CGM systems with other sensor-based wearables could open new perspectives.

### 2.3. CGM for Optimization of Athletic Performance

In competitive sports, constant optimization of physical performance is important. Wearables are already being used in competitive sports to monitor physiological variables, analyze tactical information and technical training, and to individualize training management [67,68,69]. However, wearable electrochemical sensors still only play a minor role. Yet, CGM has great potential for performance-relevant optimization.

As is the case in health care and wellness, good nutrition plays a crucial role for athletes. Numerous studies have addressed the question of nutritional strategies that can contribute to improved performance and short recovery times [70,71,72,73,74].

Pre-exercise and optimal starting conditions are a prerequisite. A well-dosed intake of carbohydrates prior to exercise can have a performance-enhancing effect [70,72,74,75,76]. This may involve replenishing glycogen stores in advance and ensuring a (subsequent) replenishment of blood glucose, thus ensuring the availability of sufficient glucose for energy metabolism [74].

During exercise, sufficient transport of glucose to the working muscles is necessary without causing supply deficits. Physiological processes of glucose regulation during exercise are very complex and dependent on several factors: blood flow through the liver and the associated hepatic glucose output, stress hormones that cause mobilization of glucose from glucose depots, uptake of blood glucose into muscle cells, food-induced increase in blood glucose, etc. [77,78]. Glucose regulation during exercise also depends on the type, duration, and intensity of physical activity [79,80].

Post-exercise, an optimal supply of nutrients as well as other actions (massage, sauna, and recovery runs) are important to accelerate recovery. CGM can provide important information on glucose conditions in all three phases.

Several CGM systems for exercise and competition management have already been applied, especially in endurance sports. A recent study investigated the relationship between running time and glucose dynamics in ultramarathon runners (n = 7) using CGM systems. The study identified a positive correlation between the lowest and average levels of Δglucose and running speed in male athletes, but not between the highest levels of Δglucose and running speed [81]. Additionally, a positive correlation was found between the amount of total carbohydrate consumption and running performance. Continuous glucose homeostasis and maintaining an appropriate glucose level seemed to be more important than achieving high glucose concentrations [81].

This is in line with the results of a case study [82]. In that study, the glucose courses of an experienced marathon runner (19 marathons, best time 3:34 h) were recorded using a CGM system during five different marathons. Different glucose patterns were observed. The best running performance was achieved with flat glucose patterns. Accordingly, these studies suggest that performance during aerobic exercise can be optimized by using CGM systems and adequate, well-timed carbohydrate intake.

Athletes must often recover as quickly as possible. CGM systems can be used to monitor the effects of intense physical exertion on glucose concentration in the recovery period. In a study with 10 athletes, both glucose dynamics after carbohydrate intake and overnight basal glucose values were altered following intense exercise to exhaustion [83]. CGM data can be used to determine when the glucose profile is normalized and returns to the pre-exercise profile. CGM systems can thus be used to notify when recovery has been completed.

## 3. Challenges and Future Perspectives

Although advancing CGM technology has led to important findings in diabetes research over the past 20 years, the many possible effects of diet, exercise, and stress on glucose regulation in metabolically healthy individuals are still largely unknown and should be explored in future CGM studies.

Another interesting area of research is how sex-specific differences in glucose metabolism affect health and physical performance. There is evidence that differences in glucose metabolism exist between men and women [84,85,86,87,88].

CGM has considerable potential to contribute to improvements in health care, well-being, physical performance, recovery, and stress even in nondiabetic individuals. To realize the full potential of CGM, careful analysis and interpretation of the collected data are necessary. In this regard, artificial intelligence (AI) and smart devices that can process large amounts of data will play a crucial role in the future.

There is already a trend toward AI in diabetes management for predicting and preventing diabetic complications [89,90,91,92]. By collecting information from the user (food intake, type of activity, etc.) and other sensors (recording heart rate, temperature, inflammatory markers, etc.), the interpretation of CGM data can be further improved.

Finally, the technical developments of CGM systems should also be mentioned. The current trend points toward less-invasive or even noninvasive systems, although they usually cannot compete yet with established devices in terms of accuracy and reliability. Some expert reviews of the technical developments and challenges are available [3,93,94,95,96,97,98,99].

One limitation of the use of current CGM systems is the inevitable time delay (mentioned in the Introduction) due to the diffusion processes of glucose. Glucose concentrations in the blood can change very rapidly, especially during high-intensity physical activity or intake of foods with high glycemic loads, resulting in possible clinically significant differences between SMBG and CGM measurement outcomes. This needs to be considered when interpreting CGM data. Additional measurements of blood glucose may provide precise clarification.

## 4. Conclusions

We observe the increasing use of wearables and interest in glucose sensors in our daily practice. CGM has a high potential for health benefits and self-optimization. However, the application possibilities of CGM systems, in our opinion, are still far from being fully exploited. To develop all fields of CGM application further and to ensure a reliable interpretation of CGM data in different fields of application, more research on glucose regulation in healthy individuals in various situations and on its influencing factors is needed.

## Data Availability

Not applicable.
